# Predictors of Functional Outcome and Mortality in Endovascular Treatment for Acute Basilar Artery Occlusion: A Single-Centre Experience

**DOI:** 10.3389/fneur.2021.731300

**Published:** 2021-10-13

**Authors:** Jie Cao, Yi Mo, Ronghua Chen, Huaming Shao, Jinggang Xuan, Ya Peng, Xucheng Zhu

**Affiliations:** Department of Neurosurgery, The First People's Hospital of Changzhou/The Third Affiliated Hospital of Soochow University, Changzhou, China

**Keywords:** acute basilar artery occlusion, endovascular treatment, functional outcome, mortality, thrombectomy

## Abstract

**Background and Purpose:** The objective of this study was to identify prognostic factors of endovascular treatment in patients with acute basilar artery occlusion and add evidence about the efficacy and safety of endovascular treatment for acute basilar artery occlusion.

**Materials and Methods:** We reviewed the data of 101 patients with acute basilar artery occlusion receiving endovascular treatment from January 2013 to September 2019. Baseline characteristics and outcomes were evaluated. A favourable functional outcome was defined as a mRS of 0 to 2 assessed at the 3 month follow-up. The association of clinical and procedural characteristics with the functional outcome and mortality was assessed.

**Results:** The study population consisted of 101 patients: 83 males and 18 females. Successful recanalization was achieved in 99 patients (97.1%). A favourable clinical outcome was observed in 50 patients (49.5%), and the overall mortality rate was 26.7%. A favourable outcome was significantly associated with NIHSS score at admission and lung infection. Mortality was associated with NIHSS score at admission, the number of thrombectomy device passes, the postoperative pons-midbrain index, and diabetes mellitus.

**Conclusions:** This study suggested that NIHSS score at admission, the number of thrombectomy device passes, the postoperative pons-midbrain index, diabetes mellitus, and lung infection can predict the functional outcome and mortality. These initial results add evidence about the efficacy and safety of endovascular treatment for acute basilar artery occlusion and need to be confirmed by further prospective studies.

## Introduction

Acute basilar artery occlusion (ABAO) is an uncommon but potentially devastating neurological condition, accounting for ~20% of posterior circulation strokes (1) and approximately 1% of all ischaemic strokes (2), and is associated with a very poor outcome (3). With variable presentations and a broad differential diagnosis (4), BAO is associated with high rates of critical disability and mortality due to injury to the brainstem. Without active intervention or treatment, the patient mortality rate in BAO can exceed 90% (5, 6).

Compared with either intravenous or intraarterial thrombolysis, systematic meta-analyses of case series and registry data have indicated that mechanical thrombectomy provides the optimal potential for improved recanalization rates and more favourable clinical outcomes in patients with BAO, but well-conducted randomised controlled trials are needed (7–13). To date, the only large randomised controlled trial (the BASICS Study) has not shown obvious advantages of endovascular therapy over medical therapy, but too long time span, recruitment without achieving anticipated and partial presence of cross-group cases weaken the objectivity of the results (14). Further evidence in the use of endovascular treatment (EVT) in posterior-circulation strokes is required.

This study is based on data from a single-centre registry with 101 patients who received EVT. endovascular treatment in patients with acute basilar artery occlusion and. The purpose of the present study was to analyse prognostic factors of clinical outcomes of endovascular treatment for acute basilar artery occlusion and add evidence about the efficacy and safety of endovascular treatment for acute basilar artery occlusion.

## Methods

### Selection of Patients

This was a retrospective study from one stroke centre in Changzhou, China. The time interval of this study ranged from January 2013 to December 2019. The clinical data of patients with ABAO confirmed by digital subtraction angiography treated with mechanical thrombectomy within 24 h from symptom onset were collected and statistically analysed.

Patients were enrolled if they met the following criteria: (a) age ≥18 years old; (b) diagnosis of BAO by CTA or MRA before EVT; (c) EVT performed within 24 h after symptom onset; and (d) mRS ≤ 2 before stroke. The exclusion criteria were as follows: (a) history of surgery or trauma within 2 months; (b) intracranial haemorrhage (ICH) or history of subarachnoid haemorrhage, tumour, ICH, or arteriovenous malformation; (c) large infarct core (exceeding two-thirds of the midbrain, pons, or either side of the cerebellum); (d) dysfunction of important organs; (e) definite bleeding tendency; and (f) voluntary abandonment of treatment or failure to follow doctor's instructions due to non-iatrogenic reasons.

All patients (or relatives) signed informed consent forms before treatment. This study was approved by the Ethics Committee of the Hospital.

### Intervention Procedure

During mechanical thrombectomy, patients were under local or general anaesthesia depending on the clinical circumstances. Each patient treated under general anaesthesia underwent tracheal intubation before anaesthesia. Oxygenation and strict blood pressure control were maintained during the procedure in all cases. All interventions were performed by senior neurointerventionalists under general or local anaesthesia. Successful recanalization after mechanical thrombectomy was defined by TICI 2b-3 (15). A 24-h follow-up non-enhanced CT was always performed to rule out complications of ICH.

In this study, the whole treatment process of mechanical thrombectomy was almost consistent with that in our previous study (16). Together with the extensive surgical experience and the progress of neurointerventional materials, the stent-retriever thrombectomy technique combined with an intermediate catheter or a direct aspiration first-pass technique (ADAPT) (17) was applied in our centre for appropriate patients.

Once atherosclerosis or dissection was considered the stroke aetiology, 10 μg/kg tirofiban was immediately administered by an intravenous bolus and continued at 0.15 μg/kg/min. Balloon angioplasty and/or stent placement was considered if there was obvious stenosis, while stent placement was used for the dissected artery.

One hundred milligrammes of aspirin and 75 mg of clopidogrel were administered at 24 h postprocedure. Tirofiban was stopped after overlapping medicine treatment for at least 6 h. Aspirin and clopidogrel were administered daily for 3 months.

### Neuroimaging Assessment

The time window was 24 h. Collected data included clinical data, such as age, sex, and vertebral artery lesions in the posterior circulation (occlusion or stenosis), cardiovascular risk factors, such as hypertension, diabetes mellitus, coronary artery disease, atrial fibrillation, history of ischaemic stroke or TIA, and smoking, stroke aetiology according to the Trial of Org 10172 in Acute Stroke Treatment (TOAST) criteria (18), and baseline NIHSS and Glasgow coma scale (GCS) scores at admission.

The baseline posterior circulation Acute Stroke Prognosis Early CT Score (Pc-ASPECTS) (19) and pons-midbrain index (PMI) (20) both at admission and within 24 h after surgery were evaluated based on preoperative and postoperative neuroimaging and CTA source images. The location of BA occlusion was also classified as proximal (from the vertebrobasilar junction to the origin of the anterior inferior cerebellar artery), middle (from the origin of the anterior inferior cerebellar artery to the origin of the superior cerebellar artery), or distal (distal to the origin of the superior cerebellar artery) according to previously published criteria (21). The OTT was defined as the time between symptom onset and the beginning of femoral artery puncture. Additionally, the puncture-to-recanalization time (PTR) was collected for reference.

The presence of hydrocephalus or evident ischaemic changes in the posterior circulation territory or ICH was reassessed via craniocerebral CT within 36 h after EVT. Symptomatic ICH was defined according to the definition of the safe thrombolysis in the stroke surveillance study (SITS-MOST): local or remote intraparenchymal haemorrhage type 2 in the 22- to 36-h follow-up imaging scans, combined with a neurological deterioration of ≥4 points on the NIHSS from baseline, from the lowest NIHSS score between baseline and 24 h, or leading to death (22). Lung infection during the perioperative period and complications related to surgical instruments and operations (new vascular occlusion, reocclusion after successful opening, ICH, arterial dissection, vasospasm, haematoma or haemorrhage at the femoral artery puncture point, hypotension requiring medication, bradycardia requiring medication, etc.), considered a potential prognostic factors, were also documented.

### Clinical Outcome Measures

The severity of stroke at the time of EVT was dichotomized as severe or mild to moderate, in conformity to the definition used in the Basilar Artery International Cooperation Study (BASICS) registry (6). Patients in a coma, with tetraplegia, or in a locked-in state were classified as having a severe stroke, whereas mild-to-moderate stroke was defined as any deficit that was less than severe. The functional outcome at 3 months was assessed with the mRS score obtained during a follow-up outpatient visit or via a semi-standardised telephone interview. A favourable outcome was defined as an mRS score of 0–2, in accordance with the definition used in the BASICS registry. Moreover, the patients were divided into two groups according to mortality to investigate the difference in the mortality rate between the two groups by risk factor stratification.

### Statistical Analysis

All statistical analyses were performed using the software package SPSS 22.0. The chi-square test or Fisher's exact test was used to compare categorical variables, and Student's *t*-test or the Mann–Whitney U test was used to compare continuous variables. A logistic regression model was used for multivariate analysis to determine whether the potential risk factors on univariate analysis remained independently associated with a favourable functional outcome and mortality at 90 days. All tests were 2-sided, and *p*-values of 0.05 or less were considered statistically significant.

## Results

### Baseline Characteristics

A total of 101 patients who underwent EVT for ABAO between January 2013 and September 2019 were included in this analysis, including 20 patients in the mild-to-moderate group and 81 patients in the severe group. Baseline clinical and neuroimaging characteristics are presented in Table 1. There were 83 males and 18 females, with a mean age of 62.2 ± 12.91 (mean ± SD) years. Eight patients (7.8%) underwent IVT prior to EVT in this series. The median baseline NIHSS score was 30 (interquartile range [IQR], 22.5–36.5), and the median total GCS score was 5 (IQR, 4–7). Among the stroke risk factors, hypertension was most prevalent (81 of 101 patients [79.4%]). In this research, atherosclerosis, accounting for 48%, remained the most common cause of ABAO.

**Table 1:** Baseline characteristics and functional outcome (mRS ≤2 vs. mRS >2).

**Characteristics**	**Overall** **(*n* = 101)**	**Outcome**		
		**mRS ≤2** **(*n* = 50)**	**mRS >2** **(*n* = 51)**	***P*-value**
Age (years), mean (SD)	62.2 (12.91)	62.5 (12.85)	62 (13.09)	0.853
Male sex, No. (%)	83 (81.4)	42 (84)	41 (80.4)	0.636
Hypertension, No. (%)	81 (79.4)	38 (76)	43 (84.3)	0.295
Diabetes mellitus, No. (%)	20 (19.6)	7 (14)	13 (25.5)	0.147
Baseline glycaemia (mmol/L), mean (SD)	8.3 (2.98)	7.84 (2.83)	8.76 (3.08)	0.065
Atrial fibrillation, No. (%)	30 (29.4)	16 (32)	14 (27.5)	0.617
Coronary heart disease, No. (%)	8 (7.8)	4 (8)	4 (7.8)	0.999
Antithrombotic treatment, No. (%)	15 (14.7)	6 (12)	9 (17.6)	0.425
History of ischaemic stroke or TIA, No. (%)	20 (19.6)	9 (18)	11 (21.6)	0.653
Smoking, No. (%)	38 (37.3)	17 (34)	21 (41.2)	0.457
GCS score at admission, median (IQR)	5 (4–7)	6 (4–9)	5 (4–6)	0.008
NIHSS score at admission, median (IQR)	30 (22.5–36.5)	26 (20–32)	35 (28–38)	<0.001
Preoperative Pc-ASPECTS, median (IQR)	9 (9–10)	9 (9–10)	9 (9–10)	0.234
Preoperative PMI, median (IQR)	0 (0–0)	0 (0–0)	0 (0–0)	0.615
IVT, No. (%)	8 (7.8)	4 (8)	4 (7.8)	0.999
Stroke aetiology, No. (%)				0.435
Atherosclerosis	49 (48.0)	21 (42)	28 (54.9)	
Cardioembolic	30 (29.4)	15 (30)	15 (29.4)	
Other	7 (29.4)	4 (8)	3 (5.9)	
Unknown	15 (14.7)	10 (20)	5 (9.8)	

*SD, standard deviation; GCS, Glasgow Coma Scale; IQR, interquartile range; Pc-ASPECTS, posterior circulation Acute Stroke Prognosis Early CT Score; PMI, pons-midbrain index; IVT, intravenous thrombolysis*.

As shown in Tables 1, 2, a favourable outcome was significantly associated with both the total GCS score at admission (*p* = 0.008) and the NIHSS score at admission (*p* < 0.001), which were also statistically associated with mortality (GCS score, *p* < 0.001; NIHSS score, *p* < 0.001), similar to diabetes mellitus (*p* = 0.039) and baseline glycaemia (mmol/L) (*p* = 0.049) (Table 2).

**Table 2:** Baseline characteristics and mortality.

**Characteristics**	**Mortality**		
	**Yes** **(*n* = 27)**	**No** **(*n* = 74)**	***P*-value**
Age (years), mean (SD)	63.9 (12.78)	61.6 (12.99)	0.451
Male sex, No. (%)	20 (74.1)	63 (85.1)	0.321
Hypertension, No. (%)	22 (81.5)	59 (79.7)	0.845
Diabetes mellitus, No. (%)	9 (33.3)	11 (14.9)	0.039
Baseline glycaemia (mmol/L), mean (SD)	9.2 (3.40)	7.97 (2.76)	0.049
Atrial fibrillation, No. (%)	10 (37)	20 (27)	0.33
Coronary heart disease, No. (%)	2 (7.4)	6 (8.1)	0.999
Antithrombotic treatment, No. (%)	4 (14.8)	11 (14.9)	0.999
History of ischaemic stroke or TIA, No. (%)	5 (18.5)	15 (20.3)	0.845
Smoking, No. (%)	13 (48.1)	25 (33.8)	0.187
GCS score at admission, median (IQR)	4 (3–6)	5.5 (4–8)	<0.001
NIHSS score at admission, median (IQR)	37 (31–39)	28 (21–35)	<0.001
Preoperative Pc-ASPECTS, median (IQR)	10 (9–10)	9 (9–9)	0.333
Preoperative PMI, median (IQR)	0 (0–0)	0 (0–0)	0.209
IVT, No. (%)	3 (11.1)	5 (6.8)	0.764
Stroke aetiology, No. (%)			0.713
Atherosclerosis	11 (40.7)	38 (51.4)	
Cardioembolic	10 (37)	20 (27)	
Other	2 (7.4)	5 (6.8)	
Unknown	4 (14.8)	11 (14.9)	

*SD, standard deviation; GCS, Glasgow Coma Scale; IQR, interquartile range; Pc-ASPECTS, posterior circulation Acute Stroke Prognosis Early CT Score; PMI, pons-midbrain index; IVT, intravenous thrombolysis*.

### Periprocedural Characteristics and Clinical Outcomes

Tables 3, 4 show the periprocedural characteristics and outcomes. The median OTT was 240 min (IQR, 180–340), and the median PTR was 65 min (IQR, 50–115). Seventy-six patients (74.5%) were treated under general anaesthesia. Compared with the pour outcome group, the PTR was significantly lower (*p* = 0.006) in the favourable outcome group, while a longer PTR seemed to be associated with a higher mortality rate (*p* = 0.002). The distal BA was the most common site of ABAO (49/101, 48%).

**Table 3:** Periprocedural characteristics and functional outcome (mRS ≤2 vs. mRS score >2).

**Characteristics**	**Overall** **(*n* = 101)**	**Outcome**		
		**mRS ≤ 2** **(*n* = 50)**	**mRS > 2** **(*n* = 51)**	***P*-value**
Symptom-onset-to-treatment time (OTT), median (IQR), min	240 (180–340)	240.5 (158.75–322.5)	240 (180–380)	0.541
OTT ≤ 360 min, No. (%)	79 (78.2)	44 (88)	35 (68.6)	0.018
Puncture-to-recanalization time (PTR), median (IQR), min	65 (50–115)	60 (45–83.25)	90 (52–120)	0.006
General anaesthesia, No. (%)	76 (74.5)	35 (70)	41 (80.4)	0.226
Number of thrombectomy device passes, median (IQR), min	1 (1–2)	1 (1–2)	2 (10–2)	0.133
BA occlusion site, No. (%)				0.807
Proximal BA	30 (29.7)	15 (30)	15 (29.4)	
Mid BA	22 (21.7)	10 (20)	12 (23.5)	
Distal BA	49 (48.6)	26 (52)	23 (45.1)	
Endovascular treatment				
IA thrombolysis, No. (%)	17 (16.7)	9 (18)	8 (15.7)	0.756
IA infusion of tirofiban, No. (%)	47 (46.1)	23 (46)	24 (47.1)	0.915
Emergency angioplasty, No. (%)				0.41
Simple stenting	11 (10.8)	5 (10)	6 (11.8)	
Simple balloon angioplasty	11 (10.8)	8 (16)	3 (5.9)	
Both stenting and balloon angioplasty	24 (23.5)	10 (20)	14 (27.5)	
None	55 (53.9)	27 (54)	28 (54.9)	
Postoperative Pc-ASPECTS, median (IQR)	6 (5–8)	7 (6–8)	6 (5–7)	0.001
Postoperative PMI, median (IQR)	2 (1–3)	1 (1–2)	3 (1–4)	0.001
Complications, No. (%)				
Complications related to surgical instruments and operations	11 (10.8)	4 (8)	7 (13.7)	0.356
Symptomatic ICH	5 (4.9)	0 (0)	5 (9.8)	0.07
Hydrocephalus	2 (2)	0 (0)	2 (3.9)	0.484
Lung infection	50 (49)	14 (28)	36 (70.6)	<0.001
TICI 2b-3, No. (%)	99 (98.0)	50 (100)	49 (96.1)	0.157

*IQR, interquartile range; Pc-ASPECTS, posterior circulation Acute Stroke Prognosis Early CT Score; BA, basilar artery; IA, intraarterial; PMI, pons-midbrain index; ICH, intracranial haemorrhage*.

**Table 4:** Periprocedural characteristics and mortality.

**Characteristics**	**Overall** **(*n* = 101)**	**Mortality**		
		**Yes** **(*n* = 27)**	**No** **(*n* = 74)**	***P*-value**
Severe group, No. (%)	81 (79.4)	25 (92.6)	56 (75.7)	0.048
Symptom-onset-to-treatment time (OTT), median (IQR), min	240 (180–340)	250 (200–386)	240 (153.75–330)	0.182
OTT ≤360 min, No. (%)	79 (78.2)	17 (63)	62 (83.8)	0.025
Puncture-to-recanalization time (PTR), median (IQR), min	65 (50–115)	93 (64–134)	60 (45–90)	0.002
General anaesthesia, No. (%)	76 (74.5)	20 (74.1)	56 (75.7)	0.869
Number of thrombectomy device passes, median (IQR), min	1 (1–2)	2 (1–3)	1 (1–2)	0.085
BA occlusion site, No. (%)				0.186
Proximal BA	30 (29.7)	7 (25.9)	23 (31.1)	
Mid BA	22 (21.8)	9 (33.3)	13 (17.6)	
Distal BA	49 (48)	11 (40.7)	38 (51.4)	
Endovascular treatment				
IA thrombolysis, No. (%)	17 (16.7)	4 (14.8)	13 (17.6)	0.979
IA infusion of tirofiban, No. (%)	47 (46.1)	11 (40.7)	36 (48.6)	0.481
Emergency angioplasty, No. (%)				0.558
Simple stenting	11 (10.8)	3 (11.1)	8 (10.8)	
Simple balloon angioplasty	11 (10.8)	1 (3.7)	10 (13.5)	
Both stenting and balloon angioplasty	24 (23.5)	8 (29.6)	16 (21.6)	
None	55 (53.9)	15 (55.6)	40 (54.1)	
Postoperative Pc-ASPECTS, median (IQR)	6 (5–8)	6 (3–8)	6 (5–8)	0.069
Postoperative PMI, median (IQR)	2 (1–3)	4 (0–6)	2 (1–3)	0.008
Complications, No. (%)				
Complications related to operations	11 (10.8)	5 (18.5)	6 (8.1)	0.26
Symptomatic ICH	5 (4.9)	5 (18.5)	0	0.001
Hydrocephalus	2 (2)	1 (3.7)	1 (1.4)	0.465
Lung infection	50 (49)	16 (59.3)	34 (45.9)	0.236
TICI 2b-3, No. (%)	99 (97.1)	25 (92.6)	74 (100)	0.119

*IQR, interquartile range; Pc-ASPECTS, posterior circulation Acute Stroke Prognosis Early CT Score; BA, basilar artery; IA, intraarterial; PMI, pons-midbrain index; ICH, intracranial haemorrhage*.

During the progression of mechanical thrombectomy, 17 patients (16.7%) were treated with mechanical thrombectomy in combination with IA thrombolysis, and 47 patients (46.1%) received an intraarterial infusion of tirofiban. Direct balloon angioplasty combined with stenting without mechanical thrombectomy was performed in eight (11.8%) patients. Angioplasty with or without stenting after mechanical thrombectomy was performed in 46 (46.1%) patients: balloon angioplasty alone in 11 patients, stent placement alone in 11 patients, and balloon angioplasty combined with stenting in 24 patients. Among all 101 patients treated with angioplasty, TICI 2b−3 recanalization was achieved in 99 patients (97.1%). No significant effect of EVT on prognosis was found.

With regard to the postoperative neuroimaging evaluation based on the Pc-ASPECTS and PMI, the median Pc-ASPECTS was 6 (IQR, 5–8), and the median PMI was 2 (IQR, 1–3). It seemed that a higher postoperative Pc-ASPECTS was statistically related to a better functional outcome (*p* = 0.001), and a lower postoperative PMI was associated with both a better functional outcome (*p* = 0.001) and a lower mortality rate (*p* = 0.008). Overall, as shown in Table 1, a favourable functional outcome (mRS score 0–2) was reached by 50 (49.5%) patients, including 13 of 20 patients (65%) in the mild-moderate group and 37 of 81 patients (45.7%) in the severe group. Mortality occurred in 25 of 27 (92.6%) patients in the severe group (*p* = 0.048). Symptomatic ICH occurred in five patients (4.9%), all of whom experienced mortality (*p* = 0.001). Other complications included complications related to surgical instruments and operations in 11 patients, hydrocephalus in two patients, and perioperative lung infection in fifty patients. Lung infection was significantly associated with a poor outcome (*p* < 0.001).

### Risk Factors for Clinical Outcomes

On multivariate logistic analysis, three clinical factors were identified as predicting good clinical outcomes, including the NIHSS score at admission (OR 0.852; 95%CI, 0.748–0.970; *p* = 0.016) and lung infection (OR 0.135; 95%CI, 0.042–0.433; *p* = 0.001). Mortality was significantly associated with the NIHSS score at admission (OR 1.186; 95%CI, 1.005–1.399; *p* = 0.044), the number of thrombectomy device passes (OR 2.612; 95%CI, 1.190–5.731; *p* = 0.017), the postoperative PMI (OR 3.222; 95%CI, 1.544–6.726; *p* = 0.002), and diabetes mellitus (OR 25.037; 95% CI, 3.449–181.750; *p* = 0.001) (Table 5).

**Table 5:** Multifactor logistic regression for functional outcome and mortality.

**Characteristics**	**Favourable outcome**		**Mortality**	
	**OR (95%CI)**	***P*-value**	**OR (95%CI)**	***P*-value**
Diabetes mellitus			25.037 (3.449–181.750)	0.001
GCS score at admission	0.898 (0.620–1.299)	0.567	0.730 (0.431–1.237)	0.242
NIHSS score at admission	0.852 (0.748–0.970)	0.016	1.186 (1.005–1.399)	0.044
OTT ≤360 min	2.509 (0.589–10.689)	0.214	0.243 (0.049–1.195)	0.082
Puncture-to-recanalization time (PTR)	0.985 (0.969–1.001)	0.059	1.016 (0.998–1.035)	0.081
Number of thrombectomy device passes			2.612 (1.190–5.731)	0.017
Postoperative Pc-ASPECTS	1.427 (0.894–2.277)	0.136	1.545 (0.841–2.837)	0.161
Postoperative PMI	0.628 (0.382–1.034)	0.067	3.222 (1.544–6.726)	0.002
Lung infection	0.135 (0.042–0.433)	0.001		

*GCS, Glasgow Coma Scale; Pc-ASPECTS, posterior circulation Acute Stroke Prognosis Early CT Score; PMI, pons-midbrain index; IVT, intravenous thrombolysis*.

## Discussion

In this study, we analysed our single-centre outcomes of EVT in patients with ABAO in the past 7 years and found that the rate of a favourable functional outcome, defined as an mRS score of 0–2 at 3 months, was 49.5%, with an overall mortality rate of 26.7%. We obtained a high recanalization rate (97.1%) in patients with BAO treated with mechanical thrombectomy.

These results are comparable to those of other series published to date of patients with BAO treated with mechanical thrombectomy in terms of the rate of successful recanalization, a favourable outcome and mortality. According to previous studies, the recanalization rate of mechanical thrombectomy, including the last generation of mechanical devices in patients with ABAO, ranged from 75 to 94.8%, with higher rates in some studies performed with accepted EVT strategies with either stent retrievers, aspiration or a combination of both. Regarding the rate of a good functional outcome (mRS 0–2) and mortality, the results ranged from 29.4 to 46.1% and 21 to 40.9%, respectively (8, 10, 12, 13, 23). Based on two separate meta-analyses, the results from our study together with those of previous reports, showing higher recanalization rates and a better prognosis for patients with ABAO managed with endovascular thrombectomy when compared with drug therapy alone (either intravenous and/or intraarterial thrombolysis), suggest that our EVT strategy (mechanical thrombectomy combined with intracranial angioplasty or IA thrombolysis or IA infusion of tirofiban) has promise in patients with ABAO (24), despite the lack of good randomised controlled trials.

In fact, whether EVT is the best treatment for ABAO is controversial in some existing retrospective studies. In a multicentre clinical registry study (the EVT for Acute Basilar Artery Occlusion Study, BASILAR), intravascular treatment within 24 h of ABAO improved the functional outcome and reduced mortality, supporting the superiority of EVT compared to drug therapy alone (25). However, according to the first randomised controlled trial to assess the effect of contemporary EVT, including stent retriever-based mechanical thrombectomy, in the treatment of acute vertebrobasilar occlusion (Endovascular treatment vs. standard medical treatment for vertebrobasilar artery occlusion (BEST): an open-label, randomised controlled trial), the difference in the occurrence of a favourable outcome between patients receiving EVT and those receiving standard medical therapy alone statistically did not make sense (26). In addition, the BASICS Study also has not shown superiority of EVT over medical therapy. All of these studies have their own limitations. BASILAR study could not balance the systematic differences between the two treatment groups. BEST study had too many cross-group individuals, leading to an insufficient study quality. The limitation of BASICS Study has been mentioned in the previous part. In our study, factors associated with EVT, such as the high recanalization rate, the course of treatment and treatment-related complications, did not affect the prognosis of BAO patients; the number of thrombectomy device passes also did not affect the prognosis and seemed to be more related to the complex vascular structure and the pathophysiology of the embolism (27). A limitation of our work is the small sample size of patients with unsuccessful reperfusion. If we can obtain more samples of unsuccessful recanalization (TICI 1-2a), the results would be more convincing. Based on the fact that a substantial proportion (~50.5%) of our patients had poor clinical outcomes (mRS score 3–6) despite TICI 2b−3 recanalization, we support that successful recanalization by EVT could be essential but not sufficient to obtain good clinical outcomes in patients with ABAO.

There is conflicting evidence on the importance of timing in treating ABAO. The current American Heart Association guidelines suggest applying mechanical thrombectomy in patients with causative occlusion of the BA by a groin puncture time within 6 h of symptom onset (class IIb; level of evidence C) (28), which is supported by several previous reports (23, 29, 30). A retrospective study of 215 patients from two endovascular centres by Bouslama et al. (31) suggests that the time to recanalization does not predict the outcome, which is in line with some other studies (13, 32). In our study, we found that a time to treatment of more than 6 h was predictive of a poor outcome, and a shorter PTR could be associated with a favourable functional outcome and a lower mortality rate, based on a single-factor analysis. However, this correlation disappeared on multivariate analysis. In addition, we found that fewer thrombectomy device passes was associated with a lower mortality rate on multivariate logistic analysis. We tentatively propose that a delay from symptom onset, which is potentially related to a poorer functional outcome, should not discourage the application of reperfusion therapy in BAO patients; in addition, factors affecting the efficiency of mechanical thrombectomy, such as a complex cerebrovascular configuration or combination with other cerebrovascular lesions and a highly viscous thrombus, may be associated with a poor functional outcome.

Several prior studies have noted the importance of clinical severity at admission and found an association between higher admission NIHSS scores and poorer clinical outcomes (23, 33). Our study confirms this finding. In addition, in contrast to a published single-centre registry (8) of 28 patients with BAO undergoing EVT, we did not find a statistically significant association between the clinical outcome and the GCS score at admission.

In the current literature, the Pc-ASPECT for the posterior circulation is the subject of discussion. Some existing studies prefer to use diffusion-weighted imaging (DWI) rather than CT for evaluation of the Pc-ASPECT (34, 35). Unfortunately, we failed to collect DWI data from most patients to test this view. As in our study, in the vast majority of patients who received both a pre-interventional and post-interventional Pc-ASPECT via CT, these scores were not predictive of the functional outcome. Although on the PMI requires CT for evaluation, the post-interventional PMI was highly predictive of mortality, as suggested by the multifactor analysis in our study. This trend is in line with other findings reported in the literature (20, 36).

In our study, diabetes mellitus was a predictor of mortality on multivariate logistic analysis, which is supported by a large single-centre report on 231 patients by Ravindren et al. (23). Most reports on this topic are not in line with this observation (11, 13, 30–33). Furthermore, we found that perioperative lung infection was predictive of an unfavourable functional outcome, despite the lack of supporting findings in the literature. In fact, lung infection is not uncommon, and most ABAO patients undergoing EVT still need prolonged bed rest, which accounts for the high probability of sputum excretion disorder and pulmonary infection. This finding could be helpful to enhance the importance of the prevention and control of perioperative lung infection.

Our study has some limitations that stem primarily from its retrospective design, small sample size, long duration, lack of randomisation and comparison with patients treated with medical management only, and heterogeneity of the centre-specific study population. Additionally, some data, including the ASITN/SIR grade, which could be important to the assessment of prognostic factors, were missing. In the future, more results from large randomised controlled trials may provide more evidence about the safety and efficacy of mechanical thrombectomy in ABAO.

## Conclusion

This study suggested that NIHSS score at admission, the number of thrombectomy device passes, the postoperative pons-midbrain index, diabetes mellitus, and lung infection can predict the functional outcome and mortality. These initial results add evidence about the efficacy and safety of endovascular treatment for acute basilar artery occlusion and need to be confirmed by further prospective studies.

## Data Availability Statement

The original contributions presented in the study are included in the article/supplementary material, further inquiries can be directed to the corresponding author/s.

## Author Contributions

YP and XZ conceived and designed the study, including quality assurance and control. JC and YM collected the data and wrote the paper. RC and JX designed the study's analytic strategy. HS reviewed and edited the manuscript. All authors read and approved the manuscript.

## Funding

This work was partly supported by Changzhou Science and Technology Support Program (No. CE20205025) and the Basic Research Project of the Changzhou Science and Technology Bureau (No. CJ20200111).

## Conflict of Interest

The authors declare that the research was conducted in the absence of any commercial or financial relationships that could be construed as a potential conflict of interest.

## Publisher's Note

All claims expressed in this article are solely those of the authors and do not necessarily represent those of their affiliated organizations, or those of the publisher, the editors and the reviewers. Any product that may be evaluated in this article, or claim that may be made by its manufacturer, is not guaranteed or endorsed by the publisher.
